# Patterns of cigarette, e-cigarette, heated tobacco, and alcohol use in solid organ transplant recipients, a pre- versus post-transplant comparison: Survey results from a transplantation center in Poland

**DOI:** 10.18332/tpc/204357

**Published:** 2025-06-10

**Authors:** Zuzanna Marczak, Bartosz Olkowski, Olga Maria Rostkowska, Dorota Miszewska-Szyszkowska, Olga Kozińska-Przybył, Tomasz Warężak, Magdalena Durlik

**Affiliations:** 1 Students’ Scientific Club, Department of Transplantation, Immunology, Nephrology and Internal Diseases, Medical University of Warsaw, Warsaw, Poland; 2 Department of Transplantation, Immunology, Nephrology and Internal Diseases, Medical University of Warsaw, Warsaw, Poland; 3 Engineering Research Institute, Universidad Cooperativa de Colombia, Medellín, Colombia

**Keywords:** alcohol, survey, transplantation, cigarettes, novel tobacco products, post-transplant behavior

## Abstract

**INTRODUCTION:**

Smoking and alcohol consumption are two harmful yet socially accepted habits in Poland. The main focus of this study was to assess patterns of tobacco and alcohol consumption in Polish transplant patients.

**METHODS:**

A survey was conducted between June and November 2023 at a transplantation center in Poland. The participants in the study were kidney, liver, or pancreas transplant recipients (aged 19–81 years). A structured questionnaire was applied to assess self-reported use of tobacco and alcohol in the pre-transplantation (pre-tx) and post-transplantation (post-tx) periods.

**RESULTS:**

Data from 215 eligible transplant recipients were analyzed. The median age was 51 years (IQR: 38.5–60.5), and 56.7% of the patients were male. Most patients (79.1%) received a kidney transplant, 20.5% a liver transplant, and 5.6% a pancreas transplant. In this cohort, tobacco use decreased by 33.7% and alcohol use decreased by 40.5% post-tx compared to pre-tx. Regarding cigarette smoking, 92% of post-tx patients did not smoke at all (vs 81.1% pre-tx). The use of e-cigarettes or heated tobacco remained relatively unchanged, with abstinence declared by 91.5% pre-tx versus 93.9% post-tx (p=0.351). When asked about alcohol consumption within the last year, 67.6% of respondents indicated that they did not consume alcohol at all (vs 50.2% pre-tx), and 26.3% had occasionally consumed alcohol (vs 40% pre-tx). More than half of the participants reported no change in their tobacco and alcohol consumption patterns (65.4% and 57.1%, respectively).

**CONCLUSIONS:**

The results of our study indicated a decrease in the use of traditional tobacco products and alcohol following transplantation. However, the use of e-cigarettes or heated tobacco remains stable and should be further examined. Therefore, it is important to develop targeted interventions to support tobacco and alcohol cessation among transplant patients.

## INTRODUCTION

Tobacco consumption is a leading cause of premature mortality in European Union countries^1^, whereas alcohol consumption accounts for >250000 deaths annually across the region^2^. Smoking cigarettes and drinking alcohol are among the most common harmful substances used, both of which can have detrimental effects on health, regardless of the amount consumed^3-5^.

Reports show that nearly a quarter of the population in the European Union smokes^1,5^. In Poland, this percentage is 27%^5^. According to the World Health Organization, there is a declining trend in smoking traditional tobacco products^3^. The expected decrease in smoking-related deaths is also noteworthy. A study by Jansen et al.^6^ projects that by 2040, the proportion of smoking-related deaths will decrease to 11% in men and 10% in women. However, this is largely true for traditional tobacco products, while data on e-cigarettes and heated tobacco are still relatively limited^3^.

Transplantation is associated with potential health risks, including side effects of immunosuppressive treatment, such as an increased risk of cardiovascular disease (CVD) or cancer^7,8^. CVDs remain the leading cause of mortality among kidney transplant patients^9,10^. Assessing the use of various recreational substances in organ transplant recipients, which further aggravates the angio-vascular and oncological risks, is crucial for evaluating their condition.

Literature indicates a link between smoking and increased risk of complications post-transplant (post-tx)^11-16^. The association between smoking and CVDs has been well studied in kidney transplant (KTX) patients^11^. Smoking further contributes to endothelial dysfunction in KTX recipients, which elevates the risk of CVDs and their consequences in the named population^12^. Studies have indicated that KTX patients who smoke are at a higher risk of graft failure, graft loss, delayed graft function, and subsequent death^13-16^.

Alcohol consumption is another risk factor that needs to be considered, especially among liver transplant (LTX) recipients. Alcohol consumption is linked to liver cirrhosis^17^, but also contributes to CVDs^18^ and elevated cancer risk^19^. Research on alcohol consumption has confirmed its harmful effects in post-KTX patients^20^. Relapse is a significant clinical issue in cases of alcohol dependence, especially among post-LTX patients. Researchers have found that increased alcohol consumption is associated with lower long-term survival rates in LTX recipients^21^.

The use of e-cigarettes or heated tobacco products has become increasingly popular, particularly among young people^22^. The long-term effects of these stimulants on the health and graft conditions of transplant patients are yet to be understood. However, it is safe to assume their toxic implications^23^ and assess their use among organ recipients for effective transplantation care.

Our study aimed to assess the patterns and changes in tobacco and alcohol consumption among transplant patients at a transplant center in Poland, and provide a basis for a more tailored health counseling approach and further research in this population.

## METHODS

### Study group and design

The cross-sectional study participants were adults who had undergone kidney, liver, or pancreas transplantation and were still transplant patients with functional grafts at the point of conducting the study. There were no other specific inclusion or exclusion criteria, except for willingness to participate in the project and verbal consent. All subjects were from a transplant center in Warsaw, Poland, which followed up on approximately 2500 patients from across the country.

Participation in this study was voluntary. The researchers provided the participants with detailed information regarding the purpose of the survey and its technical aspects. Prior to their involvement, the participants provided verbal consent. This study was acknowledged by the Bioethical Commission of the Medical University of Warsaw (MUW), decision number AKBE/227/2023. Permission to perform the study was granted by the Chief of the Department of Transplantation, Nephrology, Immunology, and Internal Diseases, MUW.

### Construction of the questionnaire

The paper questionnaire was delivered in Polish. The tool was pilot tested on a subset of participants, and the survey was adjusted based on the initial feedback and data. Our questionnaire was modified from a survey used in a study by Jodczyk et al.^24^, which explored the behavioral habits of undergraduate students during the COVID-19 pandemic.

The survey was anonymous, and no personal identification data were requested. The survey collected demographic information from the participants, including gender, height, weight, and year of birth. Furthermore, the questionnaire gathered transplant data such as the type of organ transplanted and the month and year of the most recent transplant.

In the subsequent section of the survey, participants were asked to provide a subjective assessment of the effect of transplantation on their tendency to consume tobacco and alcohol. Values from ‘-5’ to ‘-1’ indicate that the transplantation process had a negative impact; that is, the use intensified. A value of ‘0’ indicated no effect, while values from ‘+1’ to ‘+5’ indicated a positive effect of transplantation, that is, a limitation of substance use.

Further questions investigated the number of traditional cigarettes smoked per day (0, <10, 10–19, and ≥20) and the use of e-cigarettes, tobacco warmers, and waterpipes (never/several times a year/less than 20 days in 30 days/ 20 days in 30 days or more often/up to an hour a day/more than one hour a day). The full questionnaire is presented in the Supplementary file. After collecting the paper questionnaires, two researchers were tasked with transcribing all data into an Excel sheet. Statistical analyses were performed using a digitalized database.

### Distribution of the questionnaire

The research tool was a survey distributed between June and November 2023. The questionnaires were distributed in paper form among patients during their hospital stay or outpatient visits at the same center. Responses were collected by four members of the research team.

### Statistical analysis

In this study, a significance level of α=0.05 was set, accepting a 5% risk of committing a Type I error. Descriptive statistics were used to summarize the data characteristics. For continuous variables, the median was selected as the measure of central tendency given its robustness in the presence of outliers. Additionally, the interquartile range (IQR) is reported as the first (Q1) and third (Q3) quartile to provide a measure of variability within the sample. Categorical variables are presented as frequencies (n) and percentages (%). The McNemar-Bowker test was used to assess changes in paired categorical data. A *post hoc* test with adjusted p-values (p_adj_), corrected using the false discovery rate (FDR) method, further substantiates the robustness of the results. The normality of continuous data was assessed using the Shapiro-Wilk test. To assess changes in paired numeric data that exhibited deviations from normality, the Wilcoxon signed-rank test was performed. Missing values were imputed by nonparametric missing-value imputation using random forest.

Hierarchical clustering on principal components (HCPC) was employed to identify distinct substance consumption profiles among the patients in our cohort. To assess differences in the prevalence of specific characteristics within the identified clusters compared to the overall study population, the v-test was utilized.

Seventeen variables were included in the cluster analysis, encompassing key demographic factors, such as age, gender, type of organ transplanted (kidney, liver, pancreas), number of transplants, and detailed substance use patterns, both pre-transplantation (pre-tx) and post-transplantation (post-tx). Analyses were conducted using the R Statistical language (version 4.3.3) using the packages *factoextra* (version 1.0.7), *FactoMineR* (version 2.11), *report* (version 0.5.8), *correlation* (version 0.8.5), *ggstatsplot* (version 0.12.3), *gtsummary* (version 1.7.2), *ggplot2* (version 3.5.0), *missForest* (version 1.5), and *dplyr* (version 1.1.4).

## RESULTS

### Patient characteristics

Data from 215 adult patients who underwent kidney, liver, or pancreatic transplantation were analyzed. The median age of the participants was 51 years (IQR: 38.50–60.50). The sample consisted of 56.7% males and 43.3% females. The median age was 52 years (IQR: 40–62) for males and 46 years (IQR: 37–60) for females, with no statistically significant difference (p=0.074). The gender and age profiles of the patients are presented in Table 1.

**Table 1:** Gender and age profile of the participants (N=215)

*Characteristics*	*Values*
**Age** (years), median (IQR)	51.00 (38.50–60.50)
**Gender**, n (%)	
Female	93 (43.26)
Male	122 (56.74)

Table 2 presents the transplant-related characteristics of the participants, including the distribution of transplanted organs and the number of transplant procedures each patient underwent. The majority of patients (79.1%) received a kidney transplant, 20.5% had a liver transplant, and 5.6% had a pancreas transplant; 93.5% of patients had only one transplant, while 6.5% received two, i.e. the kidney and pancreas. The median time since transplantation was 60 months (IQR: 23–132).

**Table 2:** Transplant related characteristics of the participants (N=215)

*Characteristics*	*n (%)*
**Transplant**	
Kidney	170 (79.07)
Liver	44 (20.47)
Pancreas	12 (5.58)
**Number of transplants**	
1	201 (93.49)
2	14 (6.51)
**Time since last transplant** (months), median (IQR)	60.00 (23.00–132.00)

### Overall impact of transplantation on substance use

**Figure 1:**
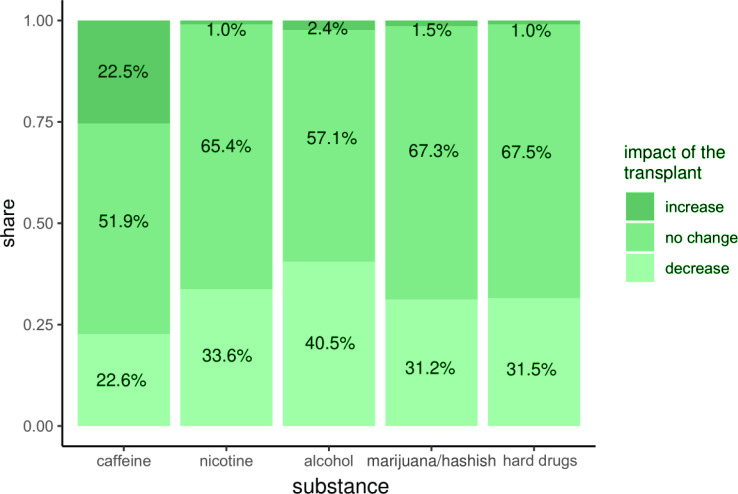
Self-assessment of the impact of transplantation on different substance use

Self-assessment of substance use post-tx indicated a general trend of reduction or stability across most substances (Figure 1). Tobacco use decreased in 33.7% of the patients post-tx, while 65.4% reported no change. This suggests that many patients reduced their use, with a minimal reported increase (1%). Alcohol consumption was also reduced in 40.5% of the patients post-tx, with 57.1% reporting no change. The self-assessment results were consistent with stratification by the following factors: kidney, liver, number of transplants, and gender, with non-significant differences in self-assessments between groups.

### Tobacco consumption: pre-tx and post-tx

The analysis in the current section provides insights into how substance use has evolved in the context of the transplantation procedure. Table 3 summarizes the frequency of daily cigarette use, e-cigarette/heated tobacco use, and alcohol use at two time points: pre- and post-tx.

**Table 3:** Tobacco and alcohol consumption pre-transplantation

*Characteristics*	*Total* *n*	*Organs transplanted*		
		*Kidney* *(N=170)* *n (%)*	*Liver* *(N=44)* *n (%)*	*Pancreas* *(N=12)* *n (%)*
**Cigarettes per day**	223			
Never smoked		136 (81.44)	35 (79.55)	11 (91.67)
<10		11 (6.59)	5 (11.36)	1 (8.33)
≥10		20 (11.98)	4 (9.09)	0 (0)
**E-cigarette and heated tobacco**	223			
Never used		153 (91.62)	40 (90.91)	11 (91.67)
Occasionally		10 (5.99)	4 (9.09)	1 (8.33)
Daily use		4 (2.40)	0 (0)	0 (0)
**Alcohol**	224			
Never		76 (45.24)	30 (68.18)	5 (41.67)
Occasionally		77 (45.83)	8 (18.18)	5 (41.67)
Once a week or more		15 (8.93)	6 (13.64)	2 (16.67)

Table 4 presents changes in the average frequency of substance consumption pre-transplantation and post-transplantation. The global test for symmetry, with p<0.001, indicates a statistically significant asymmetry in the overall distribution of cigarette consumption pre- and post-tx. Pre-tx, 81.1% of participants reported not smoking, 7.6% reported smoking <10 cigarettes per day, and 11.3% reported smoking ≥10 cigarettes daily. Post-tx, these proportions shifted to 92% reporting no smoking, 3.8% smoking <10 cigarettes per day, and 4.3% smoking ≥10 cigarettes daily.

**Table 4:** Changes in the average frequency of substance consumption pre- and post-tx

*Substance*	*Total* *n*	*Pre-tx* *(N=215)* *n (%)*	*Post-tx* *(N=215)* *n (%)*	*p*
**Cigarettes/day**	424			**<0.001**
None		172 (81.13)	195 (91.98)	
<10		16 (7.55)	8 (3.77)	
≥10		24 (11.32)	9 (4.25)	
**E-cigarette and/or heated tobacco** (e.g. IQOS, Glo, waterpipe)	425			0.351
Daily		4 (1.89)	5 (2.35)	
Never		194 (91.51)	200 (93.90)	
Occasionally		14 (6.60)	8 (3.76)	
**Alcohol**	426			**<0.001**
Never		107 (50.23)	144 (67.61)	
Occasionally		85 (39.91)	56 (26.29)	
Once a week or more		21 (9.86)	13 (6.10)	

tx: transplantation.

The reduction in smoking from high (≥10 cigarettes daily) to moderate levels (<10 cigarettes) did not occur at a statistically significant rate (p_adj_=0.248). However, the transition from ≥10 cigarettes to no smoking, post-tx (p_adj_=0.005), indicates a robust reduction in heavy smoking, with a substantial proportion of individuals who were previously heavy smokers transitioning to abstinence post-tx. Similarly, the transition from <10 cigarettes to no smoking (p_adj_=0.008) suggests that individuals who were moderate smokers pre-tx were also likely to transition to non-smoking status post-tx.

Pre-tx, 91.5% of participants reported never using e-cigarettes or heated tobacco products, 6.6% reported occasional use and 1.9% reported daily use. Post-tx, the proportion of individuals who never used these products increased slightly to 93.9%, occasional use declined to 3.8%, and daily use showed a minimal increase to 2.4%. Unlike the dynamics observed with traditional cigarette consumption, where significant reductions were noted post-tx, the use of e-cigarettes or heated tobacco appeared to remain relatively unchanged (p=0.351).

### Alcohol

Analysis of alcohol consumption patterns pre- and post-tx revealed significant changes in drinking behavior, indicating a statistically significant asymmetry in the distribution of alcohol consumption between the pre- and post-tx periods (p<0.001).

Pre-tx, 50.2% of participants reported never consuming alcohol, 40% reported occasional consumption, and 9.9% reported drinking alcohol once a week or more. Post-tx, the proportion of individuals who reported never consuming alcohol increased significantly to 67.6%, while occasional consumption decreased to 26.3%, and the proportion of individuals who consumed alcohol once a week or more decreased to 6.1%.

A significant proportion of individuals who occasionally consumed alcohol pre-tx stopped drinking altogether post-tx (p_adj_<0.001). The comparison between ‘never’ pre-tx and ‘once a week or more’ pre-tx also indicates a statistically significant shift from frequent drinking to abstinence (p_adj_=0.020), although this shift is less pronounced compared to the occasional drinkers.

The lack of significant transitions from occasional drinking to frequent drinking (p_adj_=1.000) implies that the primary behavioral shift was from drinking (occasional or frequent) to abstinence rather than a marked reduction in the frequency of drinking among those who continued to drink.

### Hierarchical clustering on principal components (HCPC)

The HCPC algorithm, which was applied to the MCA results, identified three main clusters. Cluster 1 (20.0%) consisted mostly of LTX recipients, Cluster 2 (65.6%) primarily included KTX recipients and Cluster 3 (8.4%) was characterized by males with high-risk substance use. Cluster 4 (6.0%) included double transplant recipients but is not described due to its small size and thus low relevance. Table 5 presents a detailed analysis of Clusters 1–3, highlighting the significant differences using v-tests and p-values.

**Table 5:** Description of clusters by individual category (significant results only)

*Category*	*Cla/Mod*	*Mod/Cla*	*Global*	*v-test*	p*
**Cluster 1**					
Liver transplant (yes)	95.45	97.67	20.46	13.42	**<0.001**
Kidney transplant (no)	93.33	97.67	20.93	13.22	**<0.001**
Average frequency of pre-tx alcohol consumption (none)	27.52	69.77	50.70	2.78	**0.001**
Average frequency of post-tx alcohol consumption (none)	26.71	90.69	67.91	3.78	**<0.001**
Number of transplants (one)	21.39	100.00	93.49	2.06	**0.039**
Number of transplants (two)	0.00	0.00	6.51	-2.06	**0.039**
Average frequency of pre-tx alcohol consumption (occasionally)	1.79	2.33	26.05	-4.43	**<0.001**
Average frequency of post-tx alcohol consumption (occasionally)	8.23	16.27	39.53	-3.58	**<0.001**
Kidney transplant (yes)	0.59	2.33	79.07	-13.22	**<0.001**
Liver transplant (no)	0.58	2.33	79.53	-13.42	**<0.001**
**Cluster 2**					
Liver transplant (no)	82.45	100.00	79.53	10.59	**<0.001**
Kidney transplant (yes)	82.35	99.29	79.07	10.24	**<0.001**
Number of transplants (one)	69.65	99.29	93.49	4.58	**<0.001**
Pancreas transplant (no)	68.96	99.29	94.41	4.09	**<0.001**
Average frequency of pre-tx cigarette smoking (no smoking)	71.43	88.65	81.40	3.64	**<0.001**
Average frequency of post-tx cigarette smoking (no smoking)	68.69	96.45	92.09	3.09	**0.002**
Average frequency of pre-tx e-cigarette smoking (no smoking)	69.04	96.45	91.63	3.34	**0.001**
Average frequency of post-tx e-cigarette smoking (no smoking)	68.32	97.87	93.95	3.12	**0.002**
Average frequency of pre-tx e-cigarette smoking (occasionally)	35.71	3.54	6.51	-2.29	**0.022**
Average frequency of pre-tx e-cigarette smoking (everyday)	0.00	0.00	1.86	-2.48	**0.013**
Average frequency of post-tx e-cigarette smoking (everyday)	0.00	0.00	2.32	-2.84	**0.004**
Average frequency of pre-tx cigarette smoking (≥10 cigarettes/day)	37.50	6.38	11.16	-2.92	**0.003**
Average frequency of post-tx cigarette smoking (≥10/day)	0.00	0.00	4.19	-4.06	**<0.001**
Average frequency of pre-tx alcohol consumption (occasionally)	77.64	46.81	39.53	3.02	**0.003**
Average frequency of post-tx alcohol consumption (occasionally)	80.36	31.91	26.04	2.74	**0.006**
Average frequency of post-tx alcohol consumption (once a week or more frequently)	15.38	1.41	6.04	-3.71	**<0.001**
Pancreas transplant (yes)	8.33	0.71	5.58	-4.09	**<0.001**
Number of transplants (two)	7.14	0.71	6.51	-4.58	**<0.001**
Average frequency of pre-tx alcohol consumption (once a week or more frequently)	14.28	2.13	9.76	-5.00	**<0.001**
Kidney transplant (no)	2.22	0.71	20.93	-10.24	**<0.001**
Liver transplant (yes)	0.00	0.00	20.46	-10.59	**<0.001**
**Cluster 3**					
Average frequency of pre-tx cigarette smoking (≥10/day)	54.17	72.22	11.16	6.51	**<0.001**
Average frequency of post-tx cigarette smoking (≥10/day)	100.00	50.00	4.19	6.70	**<0.001**
Average frequency of pre-tx alcohol consumption (once a week or more frequently)	47.62	55.55	9.77	5.17	**<0.001**
Average frequency of post-tx alcohol consumption (once a week or more frequently)	61.53	44.44	6.04	5.03	**<0.001**
Average frequency of pre-tx e-cigarette smoking (everyday)	100.00	22.22	1.86	4.14	**<0.001**
Average frequency of post-tx e-cigarette smoking (everyday)	60.00	16.67	2.33	2.83	**0.004**
Gender (male)	12.29	83.33	56.74	2.39	**0.017**
Gender (female)	3.22	16.67	43.25	-2.39	**0.017**
Average frequency of pre-tx e-cigarette smoking (never)	6.59	72.22	91.25	-2.52	**0.014**
Average frequency of post-tx e-cigarette smoking (never)	6.43	72.22	93.95	-3.07	**0.002**
Average frequency of pre-tx cigarette smoking (none)	1.71	16.67	81.40	-6.21	**<0.001**
Average frequency of post-tx cigarette smoking (none)	4.04	44.44	92.09	-5.67	**<0.001**
Average frequency of pre-tx alcohol consumption (never)	1.83	11.11	50.70	-3.57	**<0.001**
Average frequency of post-tx alcohol consumption (never)	3.42	27.78	67.91	-3.57	**<0.001**

Cla/Mod: the prevalence (%) of patients exhibiting a specific characteristic within the designated cluster, highlighting how common that trait is among individuals classified in that group. Mod/Cla: indicates the prevalence of the individuals within the cluster who are characterized by the studied parameter, providing insight into the distribution of that parameter among the cluster’s population. Global: the overall prevalence of the studied category across the entire sample. The v-test serves as a statistical measure that quantifies the strength of the association or difference observed between the clusters concerning the studied parameter. A higher v-test value signifies a more pronounced difference, suggesting a significant relationship between the parameter and the cluster classification. tx: transplantation.

* Statistical significance at p<0.05.

### Cluster 1: Predominantly liver transplant recipients

Cluster 1 (n=43) was primarily composed of LTX recipients (95.5%), with very few KTX recipients (2.3%). Most patients (93.5%) underwent only one transplant.

Alcohol consumption was a key characteristic. Post-tx, only 26.7% of patients in this cluster abstained from alcohol compared to 67.9% of the overall population. Pre-tx alcohol use was also more frequent, and only 27.5% had never consumed alcohol (vs 50.7% globally). The frequency of occasional post-tx alcohol use was significantly lower (16.3% vs 39.5% globally), suggesting that most patients either avoided alcohol completely or consumed it regularly. However, pre-tx occasional alcohol use was lower (2.3% vs 26.1%). The significant underrepresentation of KTX recipients (2.3% vs 79.1% globally) further reinforces that this cluster primarily comprises LTX recipients.

### Cluster 2: Predominantly kidney transplant recipients

Cluster 2 (n=141) was dominated by KTX recipients (82.4%) with a low proportion of LTX recipients (17.5%). While most patients in the global cohort had received only one transplant (93.5%), this percentage was significantly lower in Cluster 2 (69.7%). A key characteristic of this group was low tobacco and e-cigarette use. Pre-tx, 71.4% of patients had never smoked cigarettes, and 68.7% had never used e-cigarettes. Post-tx, these values remained similar (68.7% and 68.3%, respectively).

Daily e-cigarette use was almost absent in this cluster, both pre-tx (0%) and post-tx (0%), and was significantly lower than the global average (1.9% pre-tx, 2.3% post-tx). However, pre-tx heavy cigarette smoking (≥10 cigarettes/day) was reported in 6.4% of the patients, which is lower than the global prevalence of 11.2%. Importantly, none of the patients in this cluster continued to smoke at this level post-tx (0%).

### Cluster 3: Males with high-risk substance use

Cluster 3 (n=18) was distinct due to high-risk smoking and alcohol consumption behaviors. All patients in this group smoked ≥10 cigarettes per day post-tx compared to only 4.2% of the global population. Pre-tx, heavy smoking was also prevalent in this cluster (54.2% vs 11.2% overall).

Alcohol use was another defining characteristic of Cluster 3. Pre-tx, 47.6% of patients in this cluster drank alcohol at least once a week, compared to only 9.8% overall. Post-tx, this behavior intensified; 61.5% of patients continued drinking at this frequency (vs 6% globally).

The use of e-cigarettes has been alarmingly high. All patients in this cluster had used e-cigarettes daily pre-tx, and 60% continued this behavior post-tx (vs 2.3% globally).

Cluster 3 predominantly comprised male patients (83.3% vs 56.7% globally). Apart from gender, no significant associations were found with other characteristics such as age or transplanted organ type. Tobacco use was particularly concerning among individuals in Cluster 3. Pre-tx, only 16.7% of patients in this cluster had never smoked cigarettes (vs 81.4% globally). Post-tx, 44.4% remained non-smokers, well below the global rate of 92.1%. Similarly, e-cigarette abstinence was also lower. Pre-tx, only 72.2% had never used them (vs 91.3% globally), and post-tx, the rate remained the same (vs 94% globally). Alcohol consumption in Cluster 3 remained concerning. Only 11.1% of the patients in this cluster had never consumed alcohol pre-tx (vs 50.7% globally). Post-tx, 27.8% abstained, which was significantly lower than the global average of 68%.

## DISCUSSION

Transplantation is a complex and costly intervention, and continuing harmful habits such as smoking or drinking can severely undermine therapeutic outcomes. Our findings from a transplantation center in Poland indicated a statistically significant decline in tobacco and alcohol use post-tx, suggesting a positive shift in health behaviors among recipients. Traditional cigarette smoking decreased and alcohol consumption showed a marked reduction. Nonetheless, the use of e-cigarettes and heated tobacco products remained largely unchanged, highlighting the need for health intervention.

In our study, the consumption of traditional cigarettes decreased in the entire population examined. Pre-tx, 81.1% of respondents were non-smokers, and post-tx, this percentage increased to 92%. Similarly, a study by Yavuz et al.^25^ described a significant reduction in post-tx smoking in KTX recipients, with rates decreasing from 42% to 12%. This suggests that the transplant procedure or the associated lifestyle changes following medical interventions have resulted in a significant modification of smoking habits. Among the reasons for reducing smoking, the authors point to the fear of losing a transplant or frequent medical consultations^26^. Devresse et al.^27^ emphasized the importance of smoking cessation counseling and outlined key interventions, including providing non-threatening recommendations, assessing readiness to quit, offering support, and monitoring progress throughout the process.

In our research, we attempted to identify distinctive features of substance use, characterizing patient groups depending on the transplanted organ. Our data indicated that most patients in the KTX group did not smoke pre- or post-tx (71.4% pre-tx and 68.7% post-tx). This non-smoking profile in KTX patients is a positive finding, as tobacco consumption is a well-known risk factor for poor transplant outcomes, including cardiovascular complications and graft failure. Nourbala et al.^28^ in their analysis showed that smoking was a risk factor for graft loss in a population of KTX recipients. Weinrauch et al.^29^ reported that KTX recipients who smoked exhibited a 70% higher risk of mortality. Another positive outcome, in this group, is that post-tx, no patient reported smoking frequently (≥10 cigarettes/day), suggesting that the transplant experience might have encouraged cessation in KTX recipients. Kasiske and Klinger^13^ describe that efforts to promote smoking cessation pre-tx can significantly impact the patient’s post-tx survival. Other researchers have confirmed that smoking pre-tx may increase the risk of later graft loss^15,30^. In light of potential complications and the risk of transplant failure, it is crucial that pre- and post-tx care include therapeutic interventions focused on helping patients to cease smoking.

A sub-cohort of patients primarily composed of LTX recipients showed a higher frequency of alcohol consumption post-tx, with only 26.7% abstaining compared to 67.9% in the overall population. A study conducted by Koljonen et al.^31^ reported that 43% of LTX recipients consumed alcohol post-tx. In a study by Russ et al.^32^, the proportion was documented to be 27% among liver transplant recipients. Faure et al.^21^ associated reduced long-term survival post-LTX with increased alcohol consumption. Managing alcohol consumption in transplant care is crucial, particularly concerning the risk of relapse in individuals with a history of alcohol-associated liver disease pre-tx – especially given that LTX recipients often have a history of alcohol-related liver disease^33,34^. These patients may require more intensive post-tx behavioral interventions and monitoring to prevent relapse of alcohol use, considering the potential for alcohol to compromise transplant success and long-term health outcomes.

In the course of the cluster analysis, we identified a unique group of patients, characterized by high-risk substance use behaviors both pre- and post-tx. In this group which consisted mostly of men, patients smoked ≥10 cigarettes per day post-tx. Only 16.7% of the patients reported being non-smokers pre-tx, a value that increased to 44.4% post-tx. Furthermore, 60% of transplant recipients in this group reported daily use of e-cigarettes. The high prevalence of e-cigarette use, along with traditional cigarette smoking, suggests that these patients may be using e-cigarettes either as a substitute for or in conjunction with traditional cigarettes, which could increase their overall exposure to harmful substances. E-cigarettes and heated tobacco are often instinctively considered less harmful than traditional cigarettes, but recent studies indicate that smoking e-cigarettes can lead to hypertension^35^ and exacerbation of respiratory disease symptoms, including asthma and chronic obstructive pulmonary disease^36^. Therefore, examining the potential effects of these new products on transplant recipients who report smoking is a relevant research topic.

### Limitations

As with all survey-based studies, there is a potential risk of subjective reporting bias related to how participants complete the questionnaires, which may influence the accuracy of self-reported data. Nonetheless, several findings in our study showed strong statistical significance and are thus presented here and used to draw conclusions. Future studies should include objective laboratory testing, such as biomarker measurements for smoking and blood alcohol concentration for alcohol use, to validate self-reported data and reduce recall bias. The sample size of this study was relatively small (n=215); however, transplant recipients represent a small, yet continuously expanding population in Poland. This group, regardless of size, needs special consideration because of the complexity and cost of the therapeutic interventions performed, whereas addressing the topic of smoking and alcohol consumption plays a role in achieving optimal outcomes. Finally, the sample described came from a unit of the University Clinical Center of the Medical University of Warsaw which is the largest and leading transplantation center in the country.

## CONCLUSIONS

Our study showed an overall reduction in tobacco and alcohol use in patients after solid organ transplantation. A significant decrease in the use of traditional cigarettes was observed; however, the consumption of e-cigarettes and heated tobacco remained relatively stable. Liver transplant recipients require monitoring and counseling regarding alcohol consumption. Additionally, men demonstrated high-risk substance use behaviors more often. This analysis highlights the importance of an individualized approach to transplant care that addresses the unique needs of specific patient groups.

## Supplementary Material



## Data Availability

The data supporting this research are available from the authors on reasonable request.
