# Supplementary material for: Patterns of cigarette, e-cigarette, heated tobacco, and alcohol use in solid organ transplant recipients, a pre- versus post-transplant comparison: Survey results from a transplantation center in Poland
Source: Tob Prev Cessat. 2025 Jun 10;11:10.18332/tpc/204357. doi: 10.18332/tpc/204357 (PMC12150805; doi:10.18332/tpc/204357)
Supplement: Supplementary file 1 [file TPC-11-30-s1.pdf]

**Questionnaire**

# Use of tobacco products, alcohol and cannabis by patients after solid organ transplantation. Survey study..

**ENGLISH Version**

## PART 1 - Identification and transplantation questions

1. Gender:
    a. Female
    b. Male
    c. I would rather not to give it

2. Height (in cm): ……………………………………………….

3. Weight (in kg): …………………………………………………..

4. Year of birth: ………………………………………………….

5. Please indicate the organ that has been transplanted and is currently under transplant control.
    a. Kidney
    b. Liver
    c. Heart
    d. Lung
    e. Pancreas
    f. Other

6. If you chose "other" please indicate which organ was involved in the transplant.

    ……………………………………………………………………………………

7. Please provide the month and year of your last transplant.

    ………………………………………………………………………………………

## PART 2 - Stimulants

There are a few questions in front of you from the section on the use of stimulants.
Please note that the survey is anonymous and there is no possibility or need to discover your identity.

1. How would you rate the impact of transplantation on your tendency to use the following substances compared to before transplantation?
   Values of -5 to -1 indicate a decrease in use and +1 to +5 an increase in use; 0 indicates no impact.

| | -5 | -4 | -3 | -2 | -1 | 0 | 1 | 2 | 3 | 4 | 5 |
|---|---|---|---|---|---|---|---|---|---|---|---|
| caffeine consumption (coffee, tea, energy drinks, coca-cola, supplements) | | | | | | | | | | | |
| smoking or using other nicotine-containing products | | | | | | | | | | | |
| alcohol consumption | | | | | | | | | | | |
| use of cannabis THC and/or hashish | | | | | | | | | | | |
| use of hard drugs, i.e. heroin, cocaine, methamphetamine | | | | | | | | | | | |

2. On average, how many cigarettes did you smoke per day **before transplantation**? (If you do not smoke at all, please mark "0").
   a. 0
   b. <10
   c. 10-19
   d. 20-30
   e. >30

3. On average, how many cigarettes did you smoke per day **after transplantation**? (If you do not smoke at all, please mark "0").
   a. 0
   b. <10
   c. 10-19
   d. 20-30
   e. >30

4. On average, how often did you use e-cigarettes and/or heated tobacco products, i.e. IQOS, Glo or a water pipe **before transplantation?**
    a. Never
    b. Several times a year (occasionally)
    c. Less than 20 days in 30 days
    d. 20 days in 30 days or more often
    e. Daily up to a total of one hour
    f. More than one hour total every day

5. On average, how often have you used e-cigarettes and/or heated tobacco products, i.e. IQOS, glo or a water pipe **after transplantation**?
    a. Never
    b. Several times a year (occasionally)
    c. Less than 20 days in 30 days
    d. 20 days in 30 days or more often
    e. Daily up to a total of one hour
    f. More than one hour total every day

6. How often have you consumed a serving of an alcoholic beverage in the last year **before transplantation**?(1 serving (10g ethanol) is about 250ml of 5% beer, 30ml of 40% vodka or 100ml of 12% wine)
    a. I do not drink alcohol
    b. several times a year (occasionally)
    c. once a month
    d. once a week
    e. 3-4 times a week
    f. every day

7. How often have you consumed a serving of an alcoholic beverage in the last year **after transplantation**? (1 serving (10 g ethanol) is about 250 ml of 5% beer, 30 ml of 40% vodka or 100 ml of 12% wine)
    a. I do not drink alcohol
    b. several times a year (occasionally)
    c. once a month
    d. once a week
    e. 3-4 times a week
    f. every day

8. On average, how many servings of caffeinated drinks did you drink **per week before transplantation** (choice 0-50 - please write in the space provided)?

| | 0-50 |
|---|---|
| 1. espresso coffee (50 ml) | |
| 2. loose coffee/ instant coffee (200 ml) | |
| 3. coffee with milk (200 ml) | |

| | |
|---|---|
| 4. tea (200 ml) | |
| 5. energy drink (250 ml) | |
| 6. cocoa (200 ml) | |
| 7. sweet drinks containing caffeine, e.g. coca-cola (1 can/330 ml) | |

9. On average, how many servings of caffeinated drinks do you drink **per week after transplantation** (choice 0-50 - please write in the space provided)?

| | 0-50 |
|---|---|
| 1. espresso coffee (50 ml) | |
| 2. loose coffee/ instant coffee (200 ml) | |
| 3. coffee with milk (200 ml) | |
| 4. tea (200 ml) | |
| 5. energy drink (250 ml) | |
| 6. cocoa (200 ml) | |
| 7. sweet drinks containing caffeine, e.g. coca-cola (1 can/330 ml) | |

© 2025 Marczak Z. et al.
